# Evaluation of chemical castration using intra-testicular injection of zinc gluconate into the testis of the male donkey versus surgical castration: antimullerian hormone as an endpoint marker

**DOI:** 10.1186/s12917-023-03694-1

**Published:** 2023-09-02

**Authors:** Mohamed A. Hamed, Yahia A. Amin, Ragab Hassan Mohamed, Mohamed El-Adl, Shefaa M. Bazeed, Ahmed Abdou Elnegiry, Hossam Hassan Shawki, Al-Lethie A. Al-lethie

**Affiliations:** 1 Department of Surgery, Anesthesiology and Radiology, Faculty of Veterinary Medicine, Aswan University, Aswan, Egypt; 2 Department of Theriogenology, Faculty of Veterinary Medicine, Aswan University, Aswan, Egypt; 3 Department of Biochemistry and Chemistry of Nutrition, Faculty of Veterinary Medicine, Mansoura University, Mansoura, 35516 Egypt; 4 Department of Biochemistry and Chemistry of Nutrition, Faculty of Veterinary Medicine, Badr university, Badr, Cairo Egypt; 5 Department of Cytology and Histology, Faculty of Veterinary Medicine, Aswan University, Aswan, Egypt; 6 Department of Animal Genetic Resources, National Gene Bank, Giza, Egyp Egypt

**Keywords:** Testis, Castration, Donkeys, Zinc gluconate, Anti-mullerian hormone, Testosterone

## Abstract

**Background:**

Chemical castration of male animals is an alternative to surgical castration for inducing azoospermia, consequent sterility. Intra-testicular injection of zinc gluconate has been used for chemical castration in several animal species. However, its application to equine species, such as donkeys, has yet to be reported. This study aimed to evaluate the use of zinc gluconate for the chemical castration of male donkeys and to compare its effectiveness relative to routine surgical castration. For this purpose, investigations of serum testosterone and anti-Müllerian hormone levels, testicular ultrasonographic echogenicity, and histopathological findings were performed.

**Methods:**

Fourteen clinically healthy adult male donkeys were randomly and equally divided into two groups. The donkeys in group I (*n* = 7) underwent surgical castration. The donkeys in group II (*n* = 7) received intra-testicular zinc gluconate injections. The donkeys were kept under close clinical observation for 60 days. Abnormalities in donkey behavior and gross alterations in the external genitalia were recorded daily. Serum testosterone and anti-Müllerian hormone (AMH) levels were measured 15 days before the start of the treatment and 15, 30, 45, and 60 days after treatment. The testicles of group II donkeys were evaluated ultrasonographically. At the end of the study, the testes were removed and histologically examined.

**Results:**

Serum testosterone levels significantly declined compared to pre-castration levels in surgically castrated donkeys (group I), but donkeys exposed to chemical castration (group II) showed a non-significant reduction in testosterone levels. Donkeys in the surgical group had considerably lower serum AMH levels. In contrast, there was a non-significant (*p* > 0.05) increase in AMH levels in the chemical group compared with the pre-sterilization level. In addition, ultrasonographic examination revealed that the testicular echo-density had changed, as observed by a few scattered hyperechoic regions throughout the entire testis parenchyma. The histopathological investigation confirmed the presence of necrosis of the spermatogenic epithelium, increased thickness of the basement membrane of the seminiferous tubules, marked interstitial fibrosis, and shrinkage of the seminiferous tubules. Furthermore, syncytial giant cells were present in the lumen of seminiferous tubules and were associated with Sertoli cell vacuolation. Donkeys subjected to chemical castration (group II) had orchitis, as confirmed histopathologically.

**Conclusion:**

Intra-testicular injection of zinc gluconate resulted in histopathological and ultrasonographic testicular changes in adult male donkeys, which may affect their reproductive potential. However, it did not significantly alter serum testosterone or AMH levels, indicating that it cannot be used as a substitute for surgical castration in male donkeys.

## Introduction

Surgical castration is a common practice in the equine industry to sterilize animals and suppress masculine behavior. Castration may be necessary for conditions such as testicular injury, neoplasia, varicocele, hydrocele, orchitis, epididymitis, spermatic cord torsion, and inguinal hernia [1]. Although surgical castration has long been considered the gold standard for sterilization in male animals, it has several disadvantages, including being time-consuming and expensive, requiring a sterile surgical suite, anesthesia, a qualified veterinarian, the need for postoperative care, the possibility of postoperative complications, and limited application [2, 3]. Moreover, testicular removal is unacceptable in some cultures [4].

Surgical castration led to a significant decrease in post-castration serum testosterone levels, indicating that the testes are the primary source of male sex hormones. In contrast, chemical castration has varying effects on serum testosterone levels [5]. Recent studies have suggested that the anti-Müllerian hormone (AMH) is a reliable predictor of testicular tissue damage caused by many chemotherapy treatments in mice and humans [6, 7].

Chemical castration is currently considered an alternative to surgical castration because it has several advantages, such as less pain and stress, a more straightforward procedure, cost-effectiveness, and suitability for mass sterilization. Moreover, this leads to no significant complications [8]. Different substances have been used for the chemical castration of domestic animals, such as calcium chloride [5], zinc gluconate [9], and glycerol [10]. An ideal chemical agent for castration should completely prevent spermatogenesis, androgenesis, and libido without systemic toxicity or other side effects [11].

Zinc gluconate is an ideal agent for chemical castration because of its low cost, ease of use, high margin of safety without side effects, large-scale application, and permanent and irreversible results after a single treatment [12]. Intra-testicular injection of zinc gluconate has been performed in several animal species, including rats [13], cats [3], dogs [4], and monkeys [8], resulting in germ cell death, impeding spermatogenesis and leading to infertility [2].

To the best of our knowledge, there have been no previous reports of intra-testicular zinc gluconate injections in male donkeys. Hence, this study aimed to investigate the use of zinc gluconate for chemical castration in male donkeys and to compare its clinical effectiveness with that of surgical castration by assessing variations in testosterone and AMH levels, ultrasonographic changes, and histopathological findings.

## Methods

### Ethics statement

Management methods and care protocols were conducted per the guidelines of the Medical Research Ethics Committee of Mansoura University, Faculty of Veterinary Medicine, under the approved protocol number VM.R.22.10.13.

### Animals

Fourteen healthy adult donkeys (*Equus asinus*), aged 2.5 ± 1.3 years and weighing 170.0 ± 27.45 kg, were selected for evaluation from the animal house of the Veterinary Teaching Hospital at the Faculty of Veterinary Medicine, Mansoura University. The donkeys were given a mixed maintenance diet comprising 5 kg of grain, sliced wheat straw (ad libitum), and free access to water.

### Clinical examination of donkeys

All donkeys underwent a thorough clinical examination, as described by Sprayson and Thiemann [14], including assessment of vital parameters such as temperature, heart rate, and respiratory rate, to ensure their suitability for the study procedures.

### Anesthetic technique

Before surgery, all donkeys received subcutaneous prophylactic tetanus antitoxin (3000 IU). Food was withheld for 24 h, whereas water was withheld for 2 h. All donkeys were sedated with intravenous 1.1 mg/kg xylazine HCl 2% (Xyla-Ject, ADWIA Co., Egypt), and anesthesia was induced with 2.2 mg/kg ketamine HCl 5% (Ketamine, Sigma-Tec Pharmaceutical Industries, Egypt). For the surgical group (group I), anesthesia was maintained with a triple drip mixture (12.5-g guaifenesin powder, Unidrug Innovative Pharma Technologies Limited, India; 500 mg ketamine HCl 5%; and 150 mg xylazine HCl 2%) combined in 500 ml of NaCl 0.9%, administered at 1 ml/kg/h [14]. The inguinal regions of the donkeys were thoroughly examined for cryptorchidism, hernias, or scrotal scarring, which could indicate previous surgery, and all testicles were normally descended.

### Surgical procedures

The donkeys were randomly divided into two experimental groups: Group I (*n* = 7) was surgically castrated using the closed-covered castration technique [15], and Group II (*n* = 7) was chemically castrated using a single intra-testicular injection of zinc gluconate (13.1 mg/mL) neutralized by arginine (Neutersol®; Technology Transfer Inc., Columbia, MO, USA), as described by Wang [16]. Donkeys were prepared and restrained in a lateral recumbent position. The scrotal skin was scrubbed and cleaned with 70% alcohol, and the width of each testicle was measured using calipers. The injection site near the epididymal tail was disinfected with povidone-iodine 10%, and 25-gauge, 25-mm-long needles were gently inserted into the caudoventral pole of each testicle and pushed toward the dorsocranial pole. In contrast, the testicle was firmly fixed by hand against the scrotal skin. A single dose of zinc gluconate was injected into each testicle at a rate of 1 mL of solution per 2.7 cm testicle width, as described by Oliveira et al. [9]. Care was taken to avoid the seepage of the solution from the injection site.

### Clinical findings

The animals’ general behavior, scrotum changes (swelling and dermatitis), rectal temperature, presence of pain, changes in locomotion, and food and water consumption were investigated daily for the first three days. Observations were conducted weekly until the end of the 60-days study period.

### Blood collection

To estimate serum testosterone and anti-Müllerian hormone (AMH) levels in donkeys, approximately 5 ml of whole blood was collected from the jugular vein into plain labeled sample tubes under aseptic conditions. The collection was done 15 days before the start of the treatment protocol (day 0), to make sure all the donkeys were healthy, and at 15, 30, 45, and 60 days after the beginning of the experiment in both groups. The serum was separated by centrifugation (3000 rpm, 10 min), collected, and preserved at -20 °C.

#### Serum testosterone and AMH assay

Serum testosterone levels were determined using ELISA and commercial kits (BioCheck, Inc.), with the lowest detectable level of 0.05 ng/mL. Serum AMH was assayed in duplicate using the ELISA technique described by Holst et al. [17]. The AMH immunoassay test kit (AMH Gen 106 II ELISA, #A73818, Beckman Coulter, Inc., Brea, CA, USA) was utilized in the assay, with the lowest detectable level for AMH ELISA being 0.08 ng/mL. The intra-assay coefficient was 5.4%, and the inter-assay coefficient was 5.6%. The assay of both AMH and testosterone was performed using a microplate reader (Thermo Fisher Scientific) with a primary filter of 450 nm and a confirmatory wavelength of 630 nm.

### Ultrasonographic evaluation

The chemically treated group (group II) underwent transverse and longitudinal testicular ultrasonography examinations using a 7.5 MHz linear multifrequency transducer (Mindray DP-2200Vet., PR China) to image the contents of the scrotum before chemical castration by intra-testicular injection of zinc gluconate at 30 and 60 days (the end of the study).

### Histopathological evaluation

At the end of the study (60 days), the testicles of the second group were surgically excised under injectable general anesthesia (thiopental intravenous injection, 10 mg/kg BW) for histopathological evaluation. Subsequently, the donkeys were euthanized by rapid intravenous injection of thiopental sodium (1-g vial; EPICO, Egypt) at a dose of 35 mg/kg BW, as described by Abou-Khalil et al. [18].

Transverse sections of testicular tissue were grossly examined after removing tunica albicans. The testes were immediately fixed in 10% formalin for 48 h and then sliced into 1 × 1 cm pieces. The tissue was dehydrated by passing it through a graded alcohol solution. Tissue blocks were sectioned at a thickness of 4 μm using a microtome, fixed in 10% formalin, purified with methyl benzoate, and embedded in paraffin wax, which was also purified with methyl benzoate. Hematoxylin-eosin (H&E) staining was used to stain the slide specimens, and the stained testicular sections were examined under a light microscope. Pathological lesions in the testes were identified as previously described by Fox et al. [19].

### Statistical analysis

The normality of quantitative measures, including serum AMH and testosterone levels, was assessed using normal probability plots and the Kolmogorov-Smirnov test. The results are expressed as mean ± SE, and SPSS (version 23) was used for statistical analysis. An independent sample t-test was employed to investigate the statistical difference between the surgical and treated groups. The two-way ANOVA was conducted to investigate the variation over time after treatment. All values were considered statistically significant at *p* < 0.05.

## Results

### Clinical findings

**Fig. 1:**
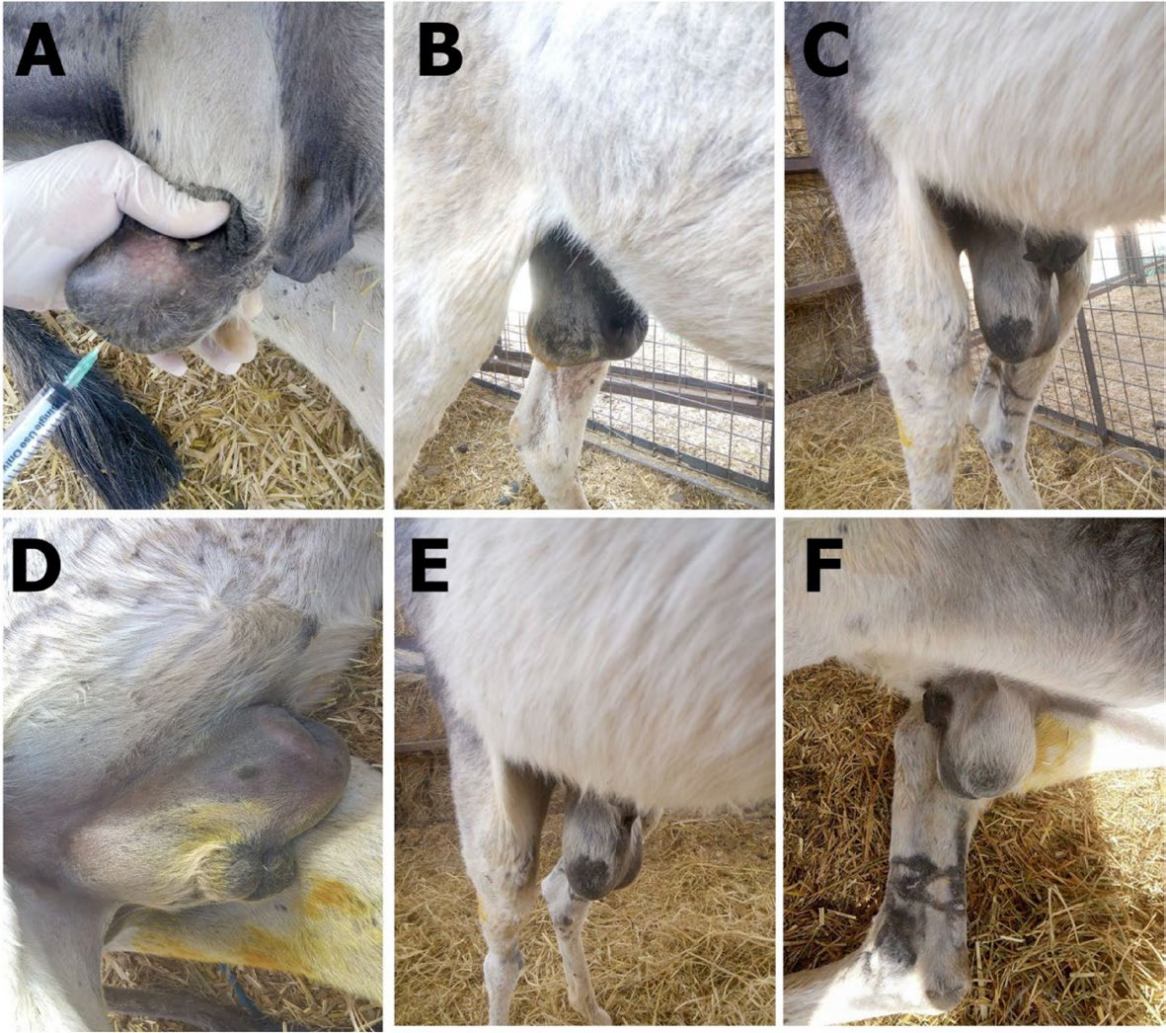
Gross changes in external genitalia in zinc gluconate treated donkeys. **A** Intratesticular injection of zinc gluconate. **B** Scrotal swelling 24 h post-intratesticular injection of drug. **C** Scrotal and preputeal swelling 48 h post injection. **D** The swelling increased severely (8th day post-injection). **E** The swelling began to decrease gradually (9^th^ day post-injection). **F** Swelling subsided completely (the 15^th^ day post-injection)

Marked testicular swelling was observed in group II donkeys 24 h after the injection (14.38 ± 0.18 cm) compared to the pre-treatment injection (13.27 ± 0.09 cm) (Fig. 1A-C). This swelling increased significantly and peaked on the 8th day after injection (23.44 ± 0.44 cm) (Fig. 1D). The swelling started to decrease on the 9th day after injection (21.77 ± 0.26 cm) (Fig. 1E). By the 15th day after injection, both the prepuce and scrotum were completely free of swelling (13.20 ± 0.08 cm) (Fig. 1F) (Table 1). Phimosis was observed in all injected cases on the third day after injection and completely subsided on the ninth day after injection; however, the urination process remained unchanged. No clinically detectable findings were observed following the injection. Animal gait, food, water consumption, and overall health remained unchanged. Donkeys in group I, which underwent closed castration procedures, showed no postsurgical complications.

**Table 1:** Measurements of testicular width (centimeter) in zinc gluconate treated donkeys (group II)

Days	Day 0	Day 1	Day 8	Day 9	Day 15
Testicular width	13.27 ± 0.09 ^a^	14.38 ± 0.18 ^a^	23.44 ± 0.44 ^b^	21.77 ± 0.26 ^b^	13.20 ± 0.08 ^a^

Values are expressed as Mean ± SE. Means carrying different superscript (a and b) are significantly different at *P* < 0.05

### Serum testosterone assay

A significant decline (*p* < 0.05) in serum testosterone concentration was observed in surgically-castrated donkeys (group I) compared to the treated group (group II) beginning on day 15 after castration (3.05 ± 0.12 and 5.5 ± 0.21 ng/ml, respectively) and decreasing to 2.90 ± 0.05 and 5.22 ± 0.32 ng/ml, respectively, by day 60.

In group I, serum testosterone levels showed a significant decrease (*p* = 0.001) at 15, 30, 45, and 60 days post-castration (3.05 ± 0.12, 3.05 ± 0.17, 2.98 ± 0.12, and 2.90 ± 0.05 ng/ml, respectively) compared to the pre-castration level (5.85 ± 0.42 ng/ml) (Table 2). In group II, serum testosterone levels were not significantly decreased (*P* > 0.05) in donkeys treated with zinc gluconate at 15, 30, 45, and 60 days (5.5 ± 0.214, 5.38 ± 0.23, 5.35 ± 0.37, and 5.22 ± 0.32 ng/ml, respectively) compared to pre-treatment values (5.77 ± 0.17 ng/ml) (Table 2).

**Table 2:** Comparison of hormonal profile in surgically castrated group compared to zinc gluconate treated group (mean ± standard error), level of testosterone and AMH test as well as optical density was stated

Parameter	Groups	Day 0	Day 15	Day 30	Day 45	Day 60
**Testosterone (ng/ml)**	Surgical group	5.85 ± 0.42 ^a^	3.05 ± 0.12 ^b A^	3.05 ± 0.17 ^b A^	2.98 ± 0.12 ^b A^	2.90 ± 0.05 ^b A^
	O.D@450 nm	1.22 ± 0.06 ^a^	1.66 ± 0.03 ^b A^	1.66 ± 0.05 ^b A^	1.67 ± 0.04 ^b A^	1.69 ± 0.012 ^b A^
	Treated group	5.77 ± 0.17	5.5 ± 0.21^B^	5.38 ± 0.23^B^	5.35 ± 0.37 ^B^	5.22 ± 0.32 ^B^
	O.D@450 nm	1.22 ± 0.02	1.25 ± 0.03 ^B^	1.26 ± 0.03 ^B^	1.26 ± 0.06 ^B^	1.28 ± 0.05 ^B^
**AMH hormone (ng/ml)**	Surgical group	12.58 ± 1.06 ^a^	3.26 ± 0.35^b A^	2.98 ± 0.14 ^b A^	2.78 ± 0.33 ^b A^	2.88 ± 0.05 ^b A^
	O.D@450 nm	0.27 ± 0.09 ^a^	1.37 ± 0.07 ^b A^	1.41 ± 0.03 ^b A^	1.11 ± 0.55 ^b A^	1.43 ± 0.01 ^b A^
	Treated group	12.91 ± 1.29	13.11 ± 1.24 ^B^	13.32 ± 1.31^B^	13.40 ± 1.17 ^B^	13.53 ± 1.43 ^B^
	O.D@450 nm	0.26 ± 0.11	0.24 ± 0.11 ^B^	0.23 ± 011 ^B^	0.22 ± 0.1 ^B^	0.21 ± 0.12 ^B^

Values are expressed as Mean ± SE. Means bearing different superscripts (small letters  of "a and b" in row and capital letters of "A and B" in column) differ significantly (*P* < 0.05)

AMH is Antimullerian hormone, O.D (optical density)

Day 0 represents the day pre-surgery in surgical group and pre-injection in treated group

### Serum AMH assay

A significant decline (*p* < 0.05) in serum AMH concentration was observed in the surgically-induced castration group (group I) (3.26 ± 0.35) compared to the treated group (group II) (13.11 ± 1.24) at day 15 after castration, which decreased further to 2.88 ± 0.05 and 13.53 ± 1.43 at day 60 in both the surgical and treated groups, respectively.

In group I, serum AMH levels were significantly decreased (*p* = 0.001) at 15, 30, 45, and 60 days post-surgery (3.267 ± 0.35, 2.98 ± 0.14, 2.78 ± 0.33, and 2.88 ± 0.05 ng/ml, respectively) compared to the pre-surgical treatment level (12.58 ± 1.06 ng/ml) (Table 2). Conversely, in group II, there was a non-significant (*p* > 0.05) increase in AMH levels at 15, 30, 45, and 60 days post-chemical sterilization (13.11 ± 1.24, 13.31 ± 1.31, 13.40 ± 1.17, and 13.53 ± 1.43 ng/ml, respectively) compared to the pre-sterilization level (12.91 ± 1.29 ng/ml) (Table 2).

### Ultrasonographic evaluation

**Fig. 2:**
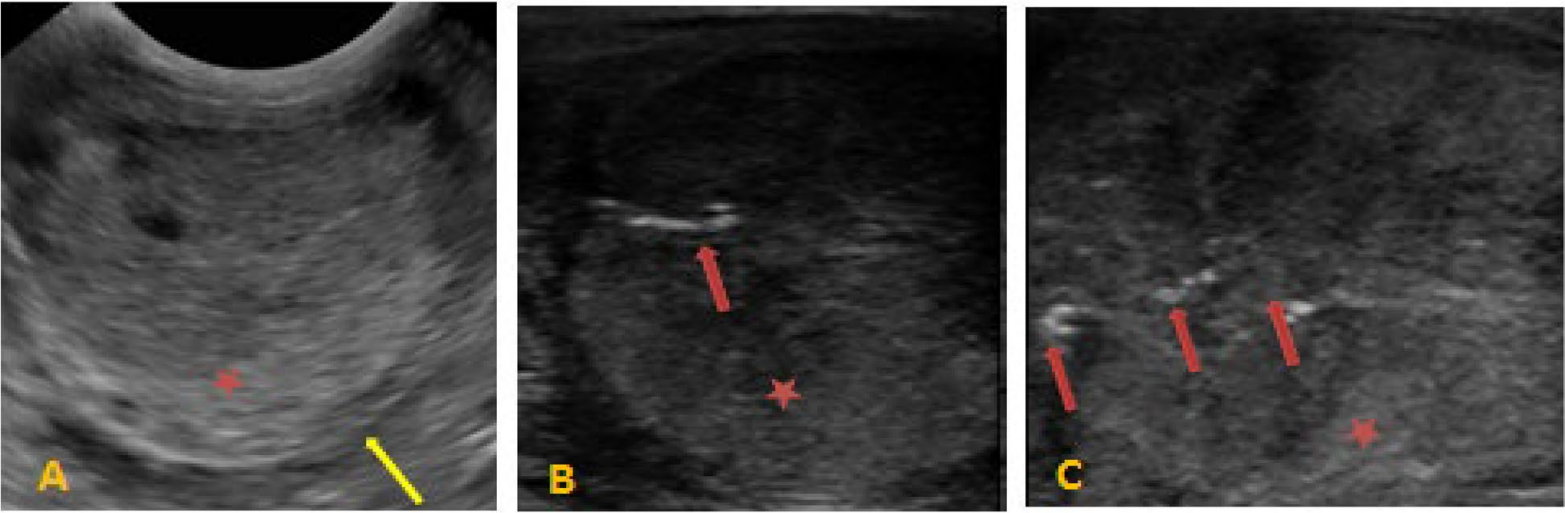
**A** Normal, homogeneous, mildly echogenic testicular parenchyma before intratesticular zinc gluconate treatment in donkey. **B** Mild changes in testicular echotexture after zinc treatment (30 days) characterized by few hyperechogenic areas (red arrows) throughout the parenchyma (red star), yellow arrows (tunica albuginea of testis). **C** Marked changes in testicular echotexture after zinc treatment (60 days) characterized by marked hyperechogenic areas (red arrows) throughout the parenchyma (red star), yellow arrows (tunica albuginea of testis)

Before intra-testicular zinc gluconate injection, ultrasonography of the scrotal contents revealed homogeneous and mildly echogenic testicular parenchyma with no abnormalities in the epididymides, testes, or spermatic cords (Fig. 2A). However, at the end of the study period (day 60), noticeable alterations in testicular echo density were observed, as evidenced by the hyperechoic regions found in scattered areas throughout the testicular parenchyma (Fig. 2B and C).

### Histopathological findings

**Fig. 3:**
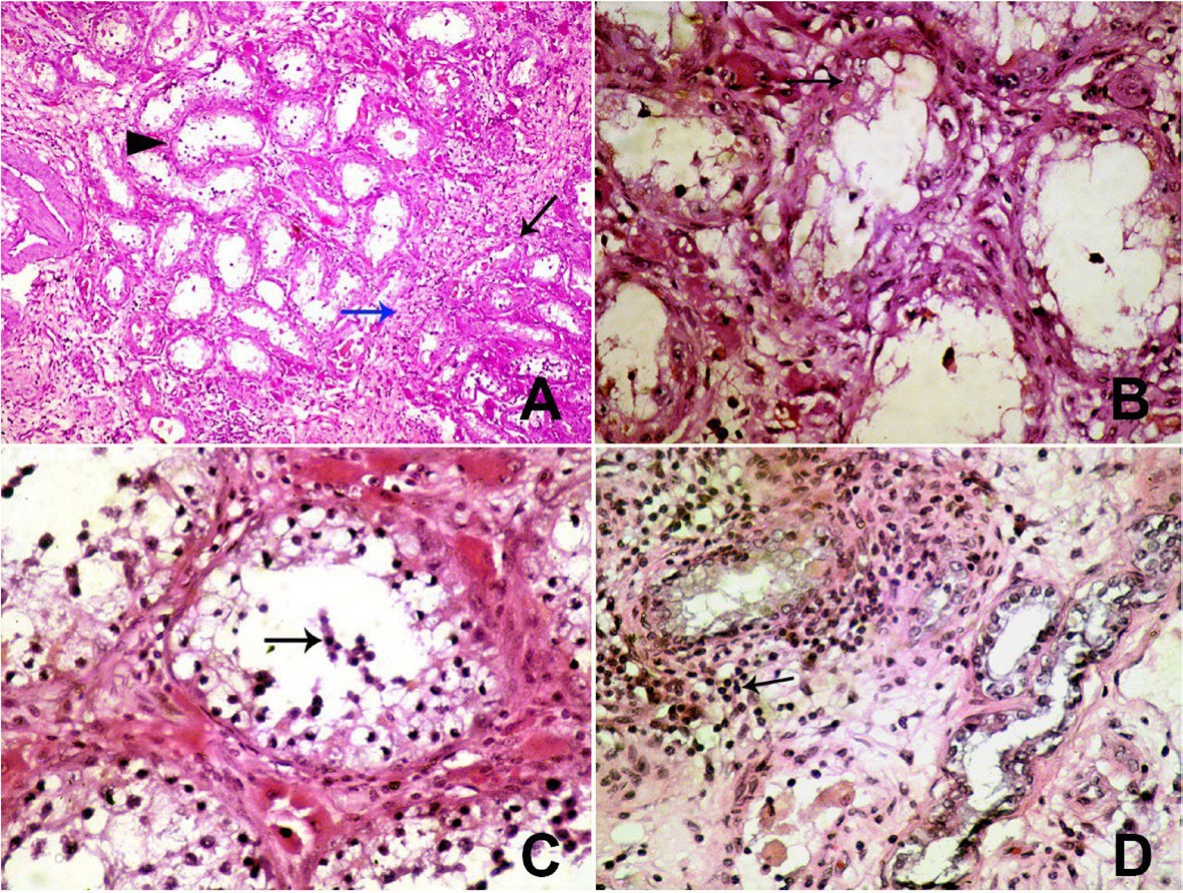
**A** Testis displays necrosis of spermatogenic epithelium, thickens of basement membrane of seminiferous tubules (arrow head), marked interstitial fibrosis (blue arrow) and shrinkage of seminiferous tubules (arrow) (HE, 100x). **B** Testis displays complete necrosis of spermatogenic epithelium and vacuolation of sertoli cells (arrow) (HE, 400x). **C** Testis displays necrosis of spermatogenic epithelium and syncytium giant cells in the lumen of seminiferous tubules (arrow) (HE, 400x) (**D**) Epididymis is showing epididymitis with lymphohistiocytic infiltrate in interstitial tissue (arrow) (HE, 100x)

Testes from treated donkeys (group II) exhibited complete necrosis of the spermatogenic epithelium, increased thickening of the basement membrane of the seminiferous tubules, marked interstitial fibrosis, and shrinkage of the seminiferous tubules (Fig. 3A). Additionally, the vacuolation of the Sertoli cells was observed (Fig. 3B), and syncytial giant cells were present in the lumen of seminiferous tubules (Fig. 3C). Furthermore, the epididymides showed epididymitis and lymphohistiocytic infiltration in the interstitial tissue (Fig. 3D).

## Discussion

Contrary to our initial prediction, intra-testicular zinc gluconate injections cannot replace surgical castration in male donkeys. The present study demonstrated that this method led to testicular alterations in the histopathology and ultrasonography of adult male donkeys, which might significantly affect their reproductive ability. However, it did not significantly alter serum testosterone or AMH levels.

In the current study, the donkeys in group I underwent a closed castration technique in which the parietal tunica vaginalis was not opened, and there was no direct access to the abdominal cavity. Open castration surgery typically requires more time and higher anesthetic doses. Additionally, there is a higher risk of spreading infection, necessitating perfect asepsis, and a hospital setting should be available for this method [15].

For a chemical castration technique to be considered a good and adequate substitute for surgical castration, several essential requirements must be satisfied, such as a high efficacy ratio and a high margin of safety in the tested animals without adverse environmental effects. Furthermore, a single dose of the chemical should efficiently achieve permanent and irreversible sterilization. Zinc gluconate is the first chemical component to meet these requirements [8]. It is used as an intra-testicular sterilant in rats [13], cats [3], dogs [4], monkeys [8], bears [20], and *Bos indicus* bull calves [21]. Therefore, this study is the first to examine intra-testicular injection of zinc gluconate in donkeys.

As intra-testicular zinc gluconate has not previously been used for chemical castration in donkeys, the dose administered in the present study was the same as that used in cats [9] and approximately twice the recommended dose for intra-testicular injection in dogs [2, 4]. According to Oliveira et al. [9], the used dose causes testicular atrophy, apparent libido reduction, and mounting aggression in cats. Furthermore, only one treated cat showed visible, mild discomfort following the procedure but responded well to symptomatic treatment.

In the current study, injection site responses, such as scrotal dermatitis or ulceration, were not observed in group II. This was likely because zinc gluconate is pH-neutral and is mainly composed of zinc, which is abundant in male reproductive fluids and tissues [22]. This finding is consistent with previous research on chemical castration in black bears [20], dogs [8, 23], and cats [9]. However, some studies on dogs have reported that zinc gluconate neutralized with arginine caused local adverse reactions in a small proportion of the tested dogs. The most common adverse reactions were scrotal necrotizing ulcers and draining sinuses. The authors attributed these adverse reactions to the injector’s lack of skill [24].

In the current study, serum testosterone levels were not significantly decreased in donkeys treated with zinc gluconate (group II) at 15, 30, 45 and 60 days post treatment. In contrast, the serum testosterone levels in group I were significantly lower in the postoperative period than in the preoperative period. These results match a previous study [8]. However, other trials in male cats [9] and dogs [25, 26] have reported that administering zinc gluconate as a permanent contraceptive through intra-testicular injection did not result in a significant difference in testosterone levels between the treated and control groups at any time point. Several studies have shown that zinc gluconate leads to irreversible fibrosis of the seminiferous tubules, testes network, and epididymis, resulting in permanent sterilization [27]. However, its toxicity is minimal in Leydig cells; hence, it stops spermatogenesis, although circulating testosterone levels are lower than normal [4, 26]. Furthermore, the chemical castration protocol involving the injection of zinc gluconate did not include orchiectomy, which meant that the source of testosterone was not fully eliminated [28]. In addition, chemical castration with CaCl_2_ in donkeys causes a longer stress reaction and greater cortisol release than surgical castration [18].

Recently, AMH was approved as a legitimate indicator of testicular damage caused by various chemotherapy treatments in mice and humans [29]. The present study revealed that the blood levels of AMH in zinc-administered donkeys started to rise after intra-testicular injection and continued to rise, reaching their peak at the end of the experiment, 60 days post-injection, when testicular degeneration occurred. These findings agree with those of Pozor et al. [29], who demonstrated that AMH concentration in the blood plasma increases in stallions with testicular degeneration (TD) caused by anti-spermatogenic or gonadotoxic substances. Furthermore, according to Murase et al., although there was no significant difference, AMH concentrations in hemi-castrated unilateral cryptorchid horses were higher than in intact horses [30]. Similar results were reported by Claes et al. [31], but the difference in this study was significant. Thus, AMH monitoring is a promising biomarker for testicular degeneration in donkeys and may be helpful in the early diagnosis of testicular degeneration.

Phimosis was observed in all injected cases on the third day after injection and subsided completely on the ninth day after injection, which is consistent with the findings of Ibrahim et al. [5], who noticed phimosis in donkeys following intra-testicular calcium chloride injection. This phimosis is usually the result of preputial edema, which leads to preputial orifice stenosis and impaired penile protrusion [32].

Histopathologically, the testes showed complete necrosis of the spermatogenic epithelium, increased thickening of the basement membrane of seminiferous tubules, marked interstitial fibrosis, and shrinkage of seminiferous tubules. In addition, the lumen of seminiferous tubules contained syncytial giant cells, and vacuolation of Sertoli cells was observed. Epididymitis with lymphocytic infiltration was observed in the interstitial tissue. These changes in the structure and function of the testes likely result in sterility, which agrees with previous studies’ results [3, 20]. Fagundes et al. [3] found that zinc gluconate injection in cats for induction of impairment of spermatogenesis as a chemical castration technique triggers an inflammatory response that reduces the diameter of the seminiferous tubules and thins the tunica propria that surrounds them.

Additionally, fibrosis may be caused by the deposition of more intertubular collagen by proliferating fibroblasts. According to Fagundes et al. [3], zinc gluconate may damage the testicles by promoting necrosis rather than inflammation. The propensity of zinc gluconate to induce injury to the testicular parenchyma, similar to that seen in autoimmune orchitis, may be a factor in testicular damage caused by compound injections. This point of view is supported by a study using Wistar rats, which showed a delay in the anti-inflammatory process after intra-testicular injection of zinc gluconate, accompanied by various anti-inflammatory medications and sodium metamizole. Since some studies have suggested that spermatogenesis recovers in men following therapy, further research is required to determine whether this occurs [33].

In our study (group II), ultrasonography of the testes subjected to zinc gluconate injection exhibited prominent changes in echo density throughout the parenchyma, with only a few isolated hyperechoic regions. These alterations in testicular echo density were supported by the observed histological abnormalities. These findings are consistent with those reported by Brito et al. [20], who noted that hyperechoic patches were occasionally observed throughout the parenchyma. In contrast, they were found only in a few dispersed locations at other times. Similarly, Cavalieri et al. [21] observed that when zinc acetate was injected into the testes using a chemical sterilization method, there was an increase in echogenicity or heterogeneity associated with diffuse or localized fibrosis and mineralization, leading to acoustic shadowing. These characteristics demonstrate the type and extent of the induced testicular alterations.

One limitation of the current study was the significant swelling in donkeys administered an intra-testicular zinc gluconate injection (group II) 24 h later, which increased markedly and continued progressing after injection. This swelling caused animal pain and induced phimosis, making semen collection impossible. Ejaculates were collected and evaluated to determine the presence of azoospermia or oligozoospermia after injection.

## Conclusion

In conclusion, our study found that intra-testicular zinc gluconate injections caused significant histopathological and ultrasonographic testicular changes in adult male donkeys, which could affect their reproductive potential. However, the injection did not significantly alter serum testosterone or AMH levels, suggesting that it cannot be substituted for surgical castration in male donkeys.

## Data Availability

This article contains all the data that was created or evaluated during the research.
